# Reranking cancer mortality using years of life lost

**DOI:** 10.1093/jncics/pkad038

**Published:** 2023-05-30

**Authors:** Cecilia Radkiewicz, Therese M-L Andersson, Jesper Lagergren

**Affiliations:** Upper Gastrointestinal Surgery, Department of Molecular Medicine and Surgery, Karolinska Institutet, Stockholm, Sweden; Department of Surgery, Capio Sankt Görans Sjukhus, Stockholm, Sweden; Department of Medical Epidemiology and Biostatistics, Karolinska Institutet, Stockholm, Sweden; Upper Gastrointestinal Surgery, Department of Molecular Medicine and Surgery, Karolinska Institutet, Stockholm, Sweden; School of Cancer and Pharmaceutical Sciences, King’s College London, UK

## Abstract

Incidence and mortality are default measures to describe cancer trends. Mortality compounds incidence and survival but not age at death. We calculated years of life lost (YLL) due to 1 of the 10 solid tumors causing most deaths (lung, colorectal, prostate, pancreatic, breast, hepatobiliary, urinary, central nervous system, gastric, melanoma) using Swedish National Cancer and Cause of Death Registers. Comparing YLL with mortality in 2019, lung (43 152 YLL) and colorectal (32 340 YLL) cancer remained at the top, pancreatic cancer was upranked fourth to third (22 592 YLL) and breast cancer fifth to fourth (21 810 YLL), while prostate cancer was downranked third to fifth (17 380 YLL). Assessing YLL over 2010-2019, women lost consistently more life years because of lung and pancreatic cancer. A downward colorectal cancer mortality trend was reflected as a YLL decline only in women. YLL is simple to calculate, is intuitive to interpret, and expands the understanding of the cancer burden on society.

Standardized incidence rates (number of incident cancer cases per person-year in the population) and mortality rates (number of cancer deaths per person-year in the population) are default measures to describe population trends in cancer, whereas cancer survival (among those diagnosed with cancer) reflects the effectiveness of cancer detection and treatment (1,2). Cancer mortality in the population is a compound measure including the number of new cancer cases and the survival among those afflicted but does not incorporate age at diagnosis, age at death, or long-term survival of palliative patients (1). There are reports of increasing early onset (younger age) cancer incidence, whereas overall rates are stable or decreasing (3-8), and younger cancer patients seem to present at a more advanced stage entailing a poorer chance of cure (8-12). This highlights a need for a more exhaustive measure to evaluate cancer trends and to make evidence-based health-care prioritizations. Years of life lost (YLL) is a measure based on the number of deaths together with age at death and has been suggested as a meaningful vital statistic reflecting disease burden in a population (13-16). We aimed to rerank the 10 solid cancers causing the largest number of cancer deaths in Sweden by incorporating the reduction in life expectancy.

We used data from the Swedish National Cancer and Cause of Death Registers and included individuals diagnosed with cancer at any age or time together with a recorded death from lung, colorectal, prostate, pancreatic, breast, hepatobiliary, urinary, central nervous system, and gastric cancer or melanoma at age 18-95 years, between years 2010 and 2019. Cancer deaths were categorized according to the 10th version of the *International Classification of Diseases* (Supplementary Table 1, available online). Publicly accessible projections of life expectancy by age (up to and including 95 years), sex, and calendar year, provided by Statistics Sweden, were used to calculate the expected age at death.

In Table 1, A, we used a traditional approach and ranked the numbers and proportions (%) of cancer deaths, mortality rates per 100 000 person-years, and male proportions of deaths in year 2019. The top 5 cancers (descending order) in terms of mortality in 2019 were lung (n = 3144; 38.7 deaths per 100 000 person-years), colorectal (n = 2543; 31.3 deaths per 100 000 person-years), prostate (n = 2063; 25.4 deaths per 100 000 person-years), pancreatic (n = 1600; 19.7 deaths per 100 000 person-years), and breast (n = 1335; 16.5 deaths per 100 000 person-years). Apart from breast cancer, only deaths from lung (46.8%) and pancreatic (49.6%) cancer were less common in men than women. The cancers were reranked using YLL (Table 1, B), calculated as the difference between age at cancer death and expected age at death for an individual of the corresponding age, sex, and calendar year in the general population and summarized by cancer site (2,13-16). Applying YLL, lung and colorectal cancer remained at the top, causing 43 152 and 32 340 YLL in 2019. Pancreatic cancer was upranked from fourth to third place (22 592 YLL) and breast cancer from fifth to fourth place (21 810 YLL), whereas prostate cancer was downranked from third to fifth place (17 380 YLL). Comparing the number of deaths and YLL in men and women, the male overbalance decreased for all non-sex–specific cancers except central nervous system, reflecting sex differences in projected life expectancy and supposedly also age at diagnosis.

**Table 1.** Traditional approach ranking the 10 most common causes of cancer death in numbers, proportion (%), mortality rate per 100 000 person-years, male proportion (%) **(A)**, and new ranking using years of life lost approach **(B)** in Sweden year 2019^a^

Cancer site	A. Traditional approach					B. Years of life lost approach		
	Rank	Deaths (%)	**Mortality rate per 100** **000**	Male %	Change in rank comparing A to B	New rank	Years of life lost (%)	Male %
Lung	1	3144 (22.5)	38.7	46.8	**→**	1	43 152 (23.3)	42.8
Colorectal	2	2543 (18.2)	31.3	54.0	**→**	2	32 340 (17.5)	53.3
Prostate	3	2063 (14.8)	25.4	100.0	**↓**	**5**	17 380 (9.4)	100.0
Pancreatic	4	1600 (11.5)	19.7	49.6	**↑**	**3**	22 592 (12.2)	48.2
Breast	5	1335 (9.6)	16.5	0.0	**↑**	**4**	21 810 (11.8)	0.0
Hepatobiliary	6	998 (7.2)	12.3	57.0	**→**	6	14 568 (7.9)	56.5
Urinary	7	740 (5.3)	9.1	70.4	**↓**	**8**	7435 (4.0)	67.8
Central nervous system	8	548 (3.9)	6.7	54.7	**↑**	**7**	11 628 (6.3)	54.7
Gastric	9	510 (3.7)	5.8	53.9	**→**	9	7159 (3.9)	53.7
Melanoma skin	10	472 (3.4)	6.3	61.9	**→**	10	6973 (3.8)	55.8

a Bold numbers indicate a shift (up- or downward) in new (years of life lost) compared to old (traditional mortality) ranking approach.

Concerning mortality trends, age-standardized (according to the population distribution in 2019) cancer mortality rates from 2010 to 2019 (Figure 1, A) revealed a decline in lung, prostate, and gastric cancer among men and breast cancer (women), as well as melanoma and colorectal cancer (both sexes, starting from approximately year 2014). Pancreatic (both sexes) and hepatobiliary (men) cancer mortality rates increased. Acknowledging that the mortality rates were age standardized and YLL were not, the latter (Figure 1, B) revealed that women lost consistently more life years than men because of lung and pancreatic cancer. The downward colorectal cancer death trend was noticeable only in women, whereas the colorectal cancer YLL increased among men. Figure 2 visualizes the overall number of YLL from 2010 to 2019, by cancer site.

**Figure 1.**
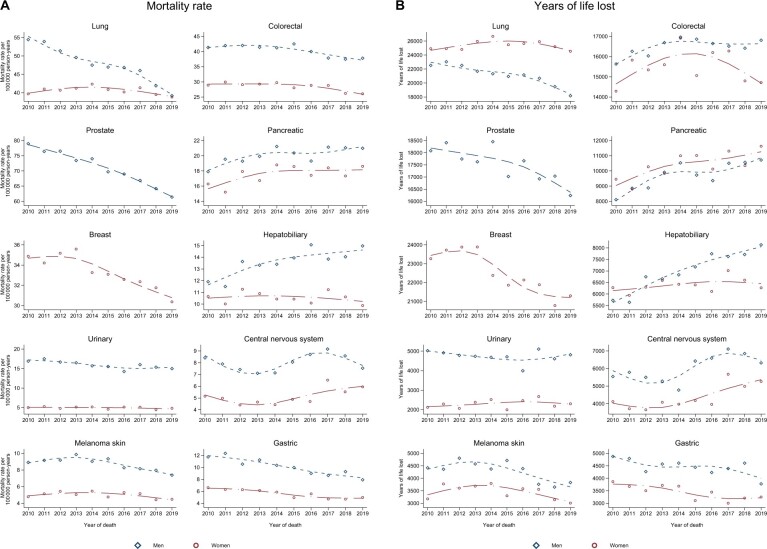
A) Age-standardized mortality rate per 100 000 person-years and **B)** years of life lost, over year of death 2010-2019 in the 10 most common causes of cancer death.

**Figure 2.**
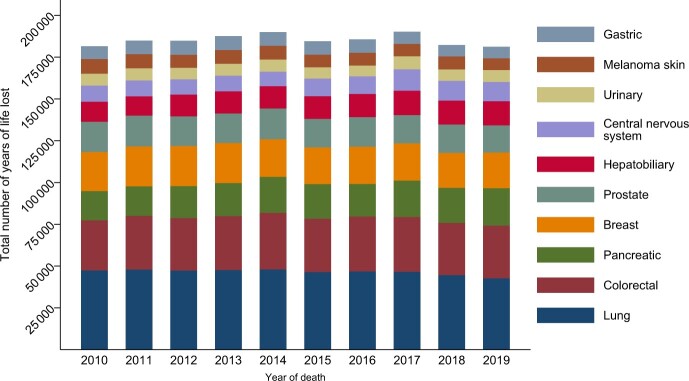
Total number of years of life lost over year of death 2010-2019 in the 10 most common causes of cancer death.

The study results are limited by the fact that poor-prognosis cancers requiring invasive diagnostic procedures for pathological confirmation are underreported in the Swedish Cancer Register (17,18). Indeed, when comparing the number of cancer deaths with a previous cancer record with publicly available death statistics published by the Swedish National Board of Health and Welfare, we missed 394 (11%) lung, 451 (22%) pancreatic, and 400 (29%) hepatobiliary cancer deaths in 2019 (Supplementary Table 2, available online) (18). Cause of death certificates can, however, both over- and underestimate cancer-specific mortality (17), and prostate cancer is probably overreported as a cause of death (19). Additional limitations include shared risk factors for cancer and death, especially in cancers associated with cigarette smoking and/or high alcohol consumption (ie, lung, pancreatic, urinary bladder, and hepatobiliary cancer). Still, the same issues apply to most population-based statistics and are not exclusive to YLL. It is, however, important to bear in mind that a population increase in itself will, despite stable incidence and mortality, result in an increasing number of deaths as well as YLL, and neither are age standardized.

Only presenting incidence, mortality, and survival as measures of cancer burden yields unfair comparisons between sites (eg, overestimation of the impact of prostate cancer on mortality) but also connotes a risk of missing clinically relevant trends (eg, a larger mortality burden of lung and pancreatic cancer among women or an increase in YLL due to colorectal cancer in men but not women). Although all vital statistics have inborn advantages and disadvantages, this study demonstrates that YLL is simple to calculate, intuitive to interpret, and a meaningful measure to be added to the statistical toolbox when presenting and comparing cancer trends within and between populations.

Informed consent is waived in this type of large-scale, register-based research. The study was approved by the Swedish Ethical Review Authority (2020-06617). All data management and analyses were performed using Stata Intercooled 17.1 (StataCorp, College Station, TX, USA; RRID: SCR_012763).

## Supplementary Material

pkad038_Supplementary_Data

## Data Availability

The cancer data were generated by the Swedish National Board of Health and Welfare, are considered sensitive, and are not publicly available due to Swedish laws and regulations. Derived, aggregated data supporting the study findings are available upon request.
