# Supplementary material for: Reranking cancer mortality using years of life lost
Source: JNCI Cancer Spectr. 2023 May 30;7(3):pkad038. doi: 10.1093/jncics/pkad038 (PMC10279998; doi:10.1093/jncics/pkad038)
Supplement: pkad038_Supplementary_Data [file pkad038_supplementary_data.pdf]

Supplementary

Cancer death classification

Cancer deaths identified from the National Cause of Death Register were categorized according to the International Classification of Diseases 10th revision (ICD-10) as presented in Supplementary Table 1. Deaths recorded as caused by cancer not otherwise specified or by metastases were re-coded as death of the most previously recorded cancer type. Deaths recorded as caused by gastrointestinal cancer not otherwise specified were re-coded as death from colorectal, pancreatic, or hepatobiliary cancer, depending on the last preceding recorded cancer diagnosis in the National Cancer Register.

Supplementary Table 1. Cancer death inclusion criteria according to version 10 of the International Classification of Diseases (ICD-10).

**Cause of death**	**ICD-10** (National Cause of Death Register)		**Cancer diagnosis**	**ICD-10** (National Cancer Register)
Lung cancer	C34.0, C34.1, C34.2, C34.3, C34.8, C34.9			
	death of unknown primary/metastases:			
	C22.9, C39.0, C39.8, C39.9, C80, C80.9	*AND*	Lung:	C34.0, C34.1, C34.2, C34.3, C34.8, C34.9
Colorectal cancer	C18.0, C18.1, C18.2, C18.3, C18.4, C18.5, C18.6, C18.7, C18.8, C18.9, C19, C20			
	death from GI cancer nos*:	*AND*	Colorectal:	C18.0, C18.1, C18.2, C18.3, C18.4, C18.5, C18.6
	C26			C18.7, C18.8, C18.9, C19.9, C20.9
	C22.9, C39.0, C39.8, C39.9, C80, C80.9	*AND*	Colorectal:	C18.0, C18.1, C18.2, C18.3, C18.4, C18.5, C18.6
				C18.7, C18.8, C18.9, C19.9, C20.9
Prostate cancer	C61			
	C22.9, C39.0, C39.8, C39.9, C80, C80.9	*AND*	Prostate:	C61.9
Pancreatic cancer	C25.0, C25.1, C25.2, C25.3, C25.4, C25.8, C25.9			
	C26	*AND*	Pancreatic:	C25.0, C25.1, C25.2, C25.3, C25.4, C25.8, C25.9
	C22.9, C39.0, C39.8, C39.9, C80, C80.9	*AND*	Pancreatic:	C25.0, C25.1, C25.2, C25.3, C25.4, C25.8, C25.9
Breast cancer	C50.0, C50.2, C50.4, C50.8, C50.9			
	C22.9, C39.0, C39.8, C39.9, C80, C80.9	*AND*	Breast:	C50.0, C50.1, C50.2, C50.3, C50.4, C50.5, C50.6, C50.8, C50.9
Hepatobiliary cancer	C17.0, C22.0, C22.1, C22.3, C22.4, C22.7, C23, C24.0, C24.1, C24.8, C24.9			
	C26	*AND*	Hepatobiliary:	C17.0, C22.0, C22.1, C23.9, C24.0, C24.1, C24.8, C24.9
	C22.9, C39.0, C39.8, C39.9, C80, C80.9	*AND*	Hepatobiliary:	C17.0, C22.0, C22.1, C23.9, C24.0, C24.1, C24.8, C24.9

Urinary cancer	C66, C67.0, C67.1, C67.2, C67.5, C67.6, C67.7, C67.8, C67.9, C68.0, C68.8, C68.9			
	C22.9, C39.0, C39.8, C39.9, C80, C80.9	*AND*	Urinary:	C66.9, C67.0, C67.1, C67.2, C67.3, C67.4, C67.5, C67.6, C67.7, C67.8, C67.9, C68.0, C68.1, C68.8, C68.9
Central nervous system cancer	C71.0, C71.1, C71.2, C71.3, C71.4, C71.5, C71.6, C71.7, C71.8, C71.9			
	C22.9, C39.0, C39.8, C39.9, C80, C80.9	*AND*	CNS:	C71.0, C71.1, C71.2, C71.3, C71.4, C71.5, C71.6, C71.7, C71.8, C71.9
Melanoma skin cancer	C43.1, C43.2, C43.3, C43.4, C43.5, C43.6, C43.7, C43.8, C43.9			
	C22.9, C39.0, C39.8, C39.9, C80, C80.9	*AND*	Melanoma skin:	C43.0, C43.1, C43.2, C43.3, C43.4, C43.5, C43.6, C43.7, C43.8, C43.9
Gastric cancer	C16.0, C16.1, C16.2, C16.3, C16.4, C16.8, C16.9			
	C26	*AND*	Gastric:	C16.0, C16.1, C16.2, C16.3, C16.4, C16.5, C16.6, C16.8, C16.9
	C22.9, C39.0, C39.8, C39.9, C80, C80.9	*AND*	Gastric:	C16.0, C16.1, C16.2, C16.3, C16.4, C16.5, C16.6, C16.8, C16.9

*Gastrointestinal cancer not otherwise specified

A quality control comparing the number of deaths in 2019 in our data (which required a preceding cancer record in the National Cancer Register) with the number of reported deaths from the Swedish National Board of Health and Welfare, confirmed an acceptable concordance for most sites except for pancreatic and hepatobiliary cancer were 451 (22%) and 400 (29%) of cancer deaths respectively were missing (Supplementary Table 2).

Supplementary Table 2. Comparing the number of cancer deaths with a previously recorded cancer at age 18-94 years with the reported number of deaths according to ICD-10 from the Swedish National Board of Health and Welfare, age at death 20-94, in year 2019.

		**N deaths**		**Absolute difference**	
**Cancer site**	**ICD-10**	**Registered cancer**	**Reported death***	**n**	**%**
Lung	C34	3144	3538	-394	-11
Colorectal	C18-C20	2543	2597	-54	-2
Prostate	C61	2063	2129	-66	-3
Pancreatic	C25-C26	1600	2051	-451	-22
Breast	C50	1335	1309	26	2
Hepatobiliary	C17, C22-C24	998	1398	-400	-29
Urinary	C66-C68	740	767	-27	-4
Central nervous system	C71	548	605	-57	-9
Melanoma skin	C16	472	540	-68	-13
Gastric	C43	510	464	46	10

*https://sdb.socialstyrelsen.se/if_dor/val.aspx
